# Survival of mature T cells depends on signaling through HOIP

**DOI:** 10.1038/srep36135

**Published:** 2016-10-27

**Authors:** Kazumi Okamura, Akiko Kitamura, Yoshiteru Sasaki, Doo Hyun Chung, Shoji Kagami, Kazuhiro Iwai, Koji Yasutomo

**Affiliations:** 1 Department of Immunology & Parasitology, Graduate School of Medicine, Tokushima University, Tokushima, Japan; 2 Department of Pediatrics, Graduate School of Medicine, Tokushima University, Tokushima, Japan; 3 Department of Molecular and Cellular Physiology, Graduate School of Medicine, Kyoto University, Kyoto, Japan; 4 Department of Pathology, Seoul National University College of Medicine, Seoul, Korea; 5 Core Research for Evolutional Science and Technology, Japan Agency for Medical Research and Development, Tokyo, Japan

## Abstract

T cell development in the thymus is controlled by a multistep process. The NF-κB pathway regulates T cell development as well as T cell activation at multiple differentiation stages. The linear ubiquitin chain assembly complex (LUBAC) is composed of Sharpin, HOIL-1L and HOIP, and it is crucial for regulating the NF-κB and cell death pathways. However, little is known about the roles of LUBAC in T-cell development and activation. Here, we show that in T-HOIP^Δlinear^ mice lacking the ubiquitin ligase activity of LUBAC, thymic CD4^+^ or CD8^+^ T cell numbers were markedly reduced with severe defects in NKT cell development. HOIP^Δlinear^ CD4^+^ T cells failed to phosphorylate IκBα and JNK through T cell receptor-mediated stimulation. Mature CD4^+^ and CD8^+^ T cells in T-HOIP^Δlinear^ mice underwent apoptosis more rapidly than control T cells, and it was accompanied by lower CD127 expression on CD4^+^CD24^low^ and CD8^+^CD24^low^ T cells in the thymus. The enforced expression of CD127 in T-HOIP^Δlinear^ thymocytes rescued the development of mature CD8^+^ T cells. Collectively, our results showed that LUBAC ligase activity is key for the survival of mature T cells, and suggest multiple roles of the NF-κB and cell death pathways in activating or maintaining T cell-mediated adaptive immune responses.

T cells express the T cell receptor (TCR) that recognizes a peptide presented by the MHC. T cells subsequently differentiate toward various effector cells that are required for combating microorganisms or tumor cells^1^,^2^,^3^,^4^. Importantly, excessive activation of effector T cells can lead to various diseases including autoimmune disorders^5^.CD4^+^CD8^+^ cells in the thymus receive TCR signals and the quantity or the quality of TCR signaling dictates the differentiation to mature CD4^+^ or CD8^+^ T cells^6^,^7^,^8^. Th-POK and RUNX3 are crucial transcription factors modulating the lineage differentiation to CD4^+^ or CD8^+^ T cells, respectively^9^,^10^,^11^,^12^. The relationship between TCR signaling and transcriptional regulation remains unclear. In the thymus, the differentiation of T cells beyond the CD4^+^CD8^+^ cell stage requires persistent TCR signaling^13^,^14^. Moreover, IL-7 receptor signaling is crucial for the final maturation or survival of CD4^+^ and CD8^+^ T cells in the thymus^15^,^16^.

The NF-κB family includes five related proteins, c-Rel, p65, RelB, p50 and p52. Those proteins form homodimers and heterodimers in specific combinations together with a regulatory protein, the inhibitor IκB^17^. A variety of extracellular signals engage the NF-κB pathway through signaling networks that converge on the IκB kinase (IKK) complex comprised of IKKα and IKKβ together with a regulatory protein, IKKγ (NEMO). The phosphorylation of IKKβ leads to the phosphorylation of IκB, triggering the polyubiquitination and subsequent degradation of IκB, allowing NF-κB dimers to translocate to the nucleus. The NF-κB pathway plays important roles in T cell development and inflammatory responses. When thymocytes are conditionally deficient for NEMO, the mice produced far fewer (<10%) mature CD4^+^ and CD8^+^ T cells in the spleen than did control mice^18^. The deficiency of IKKβ reduced the number of mature T cells in the spleen to 20–50% of those in control mice^18^. However, the specific roles of the distinct NF-κB family members in thymocyte differentiation and maturation following TCRαβ repertoire selection remain poorly defined. In this regard, ubiquitin chains are assembled by the linear ubiquitin chain assembly complex (LUBAC). This complex constitutes a regulatory unit of the NF-κB pathway, contributing to its activation^19^,^20^,^21^,^22^. LUBAC is composed of three proteins, HOIP (*Rnf31*), HOIL-1L (*Rbck1*) and Sharpin^20^,^21^. LUBAC-mediated ubiquitination of NEMO activates NF-κB with linear ubiquitin chains, which is required for IKKβ phosphorylation, resulting in degradation of IκBα. One paper reported that an inherited mutation in HOIP caused multi-organ autoinflammation, combined immunodeficiency, subclinical amylopectinosis and systemic lymphangiectasia^23^. However, the precise role of HOIP or LUBAC ligase activity in T cell development is poorly understood.

Here, we demonstrated that T cell-specific T-HOIP^Δlinear^ mice showed impairments of mature T cell development and proliferative responses. Those data highlighted the HOIP-mediated NF-κB pathway as a crucial pathway in the regulation of T cell development. Furthermore, our data indicated that deficiency of LUBAC ligase activity disturbed the development of mature T cells and their function, suggesting the important role of LUBAC for T cell-mediated adaptive immune responses.

## Results

### The deficiency of Rnf-31 ligase activity in T cells impaired the development of mature T cells in the thymus

**Figure 1:**
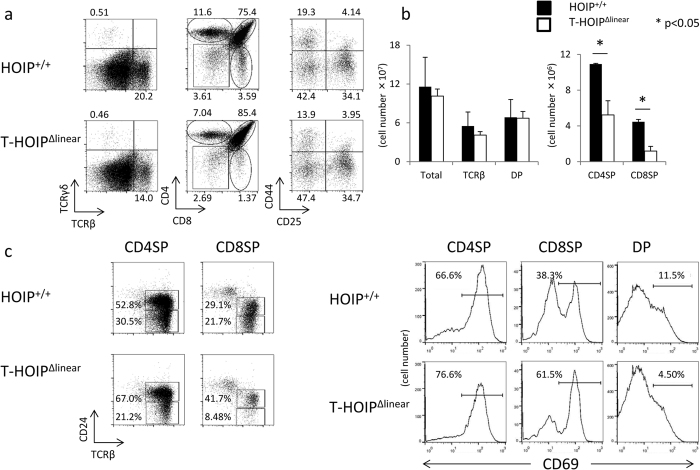
HOIP ligase activity is required for development of CD4^+^ or CD8^+^ T cells. (**a**) Thymocytes from T-HOIP^Δlinear^ mice and HOIP^+/+^ mice were stained with anti-CD4, anti-CD8α, anti-CD25, anti-CD44, anti-TCRβ and anti-TCRγ antibodies and their frequencies were evaluated by flow cytometry. The panels of TCRβ/TCRγ and CD4/CD8 were gated on lymphocytes in an FSC/SSC gate. The panel of CD44/CD25 was gated on CD4^−^CD8^−^ cells. The number indicates the percentage of each population in the viable cell fraction. (**b**) Absolute numbers of total thymocytes, TCRβ^+^ cells, CD4^+^CD8^+^ cells (DP), CD4^+^CD8^−^ (CD4SP) and CD4^−^CD8^+^ (CD8SP) cells from T-HOIP^Δlinear^ (open) or HOIP^+/+^ (filled) mice at 8 weeks of age are shown. Data are shown as means ± SEM. *p < 0.05 (**c**) Thymocytes from T-HOIP^Δlinear^ mice or HOIP^+/+^ mice were stained with anti-CD4, anti-CD8α, anti-TCRβ, anti-CD24 and anti-CD69 antibodies and the frequencies of cells expressing CD24/TCRβ or CD69 were determined by flow cytometry using gates for CD4^+^CD8^+^ (DP), CD4^+^CD8^−^ (CD4SP) and CD4^−^CD8^+^ (CD8SP) cells. The number indicates the percentage of CD69^+^ cells in the DP, CD4SP and CD8SP cell fractions. The data in these figures are representative of four independent experiments.

To evaluate the involvement of HOIP in T cell development, we established *Rnf31*^Δlinear/Δlinear^ mice with a CD4-*Cre* transgene (T-HOIP^Δlinear^ mice). The frequency of TCRβ^+^ cells in the thymus was reduced in T-HOIP^Δlinear^ mice and the relative and absolute numbers of CD4^+^CD8^−^ and CD4^−^CD8^+^ cells were markedly reduced in T-HOIP^Δlinear^ mice whereas CD4^+^CD8^+^ cells were not depressed (Fig. 1a,b). The effect was much stronger in CD4^−^CD8^+^ cells than CD4^+^CD8^−^ cells. The frequency of TCRβ^+^ cells in T-HOIP^Δlinear^ mice was equivalent to that of *Rnf31*^+/+^ mice with CD4-*Cre* transgene (HOIP^+/+^) mice (Fig. 1a). Mature CD4^−^CD8^+^ cells and CD4^+^CD8^−^T cells in the thymus downregulate CD24 and CD69 during the final maturation steps^15^. T-HOIP^Δlinear^ mice had relatively higher frequencies of CD24-positive and CD69-positive cells in both CD4^+^CD8^−^TCRβ^+^ and CD4^−^CD8^+^TCRβ^+^ fractions than did HOIP^+/+^ mice (Fig. 1c). These results suggested that HOIP-mediated ligase activity was required for final maturation or survival of mature CD4^+^CD8^−^ and CD4^−^CD8^+^ T cells in the thymus.

### T-HOIP^Δlinear^ mice had lower numbers of mature T cells in the spleen and lymph nodes

**Figure 2:**
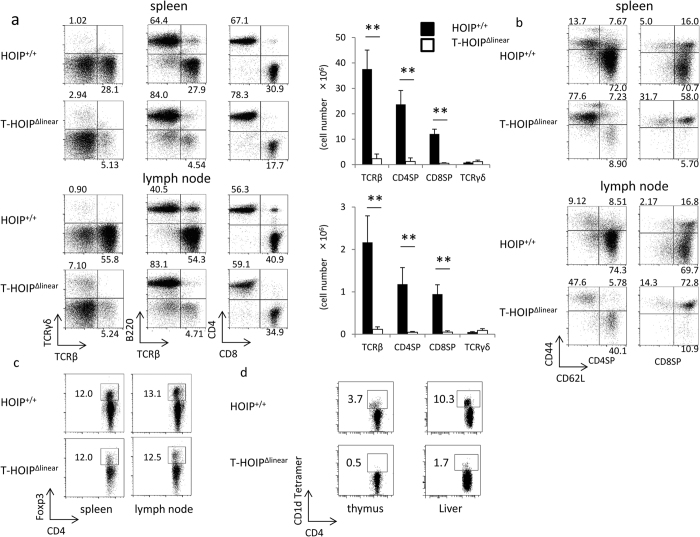
Marked decrease of CD4^+^ or CD8^+^ T cells in T-HOIP^Δlinear^ mice. (**a**) Spleen cells from T-HOIP^Δlinear^ mice and HOIP^+/+^ mice were stained with anti-CD4, anti-CD8α, anti-TCRβ, anti-TCRγ and anti-B220 antibodies and the frequencies of cells expressing TCRβ/TCRγ, TCRβ/B220 and CD4/CD8 were evaluated by gating on lymphocytes in an FSC/SSC gate. The number indicates the percentage of each population within the viable population (left and middle panels) and the percentage of each population in the TCRβ^+^ population (right panel). Data show absolute numbers of total thymocytes, TCRβ^+^ cells, TCRγ^+^, CD4^+^CD8^−^ (CD4SP) and CD4^−^CD8^+^ (CD8SP) cells from T-HOIP^Δlinear^ (open) or HOIP^+/+^ (filled) mice at the age of 8 weeks. Data are presented as means ± SEM. **p < 0.01. Spleen cells or liver lymphocytes from T-HOIP^Δlinear^ or HOIP^+/+^ mice were stained with a combination of (**b**) anti-CD4, anti-CD8α, anti-CD44 and anti-CD62L antibodies, or (**c**) anti-CD4, anti-TCRβ and anti-Foxp3 or (**d**) anti-CD4 and anti-CD1d tetramer. The frequency of CD44/CD62L cells was evaluated by flow cytometry by gating on CD4^+^CD8^−^ (CD4SP) or CD4^−^CD8^+^ (CD8SP), or CD4/Foxp3 using a primary FSC/SSC gate to identify lymphocytes expressing CD4/CD1d. The number indicates the percentage of each population within the viable population. The data in these figures are representative of four independent experiments.

We next assessed the T cell numbers and phenotypes in the spleen and lymph nodes of T-HOIP^Δlinear^ mice. The relative frequencies of TCRβ^+^ to TCRγ^+^ cells or TCRβ^+^ cells to B220^+^ cells was markedly reduced in the spleen and lymph nodes of T-HOIP^Δlinear^ mice (Fig. 2a). The total cell numbers of TCRβ^+^, CD4^+^ and CD8^+^ T cells in the spleen and lymph nodes in T-HOIP^Δlinear^ mice were also much less than in control mice (Fig. 2a). The relative frequency of CD8^+^ cells to CD4^+^ cells was reduced in HOIP^−/−^ mice in the spleen (Fig. 2a). T-HOIP^Δlinear^ mice possessed higher numbers of CD44^hi^CD62L^lo^CD4^+^ and CD44^hi^CD62L^lo^CD8^+^ T cells compared with control mice (Fig. 2b), suggesting that mature T cells from T-HOIP^Δlinear^ mice had undergone activation after being exported from the thymus. The relative frequency of CD4^+^Foxp3^+^ regulatory T cells in CD4^+^ T cells was not affected in the lymph nodes of T-HOIP^Δlinear^ mice (Fig. 2c), whereas the frequency of CD4^+^CD1d tetramer^+^ NKT cells was reduced in the thymus and liver of T-HOIP^Δlinear^ mice (Fig. 2d). Taken together, those data demonstrated that HOIP-deficiency in T cells markedly impaired the differentiation or survival of both mature CD4^+^ and CD8^+^ T cells with striking defects in the development of NKT cells.

### CD4^+^ T cells in T-HOIP^Δlinear^ mice proliferated poorly after TCR ligation

**Figure 3:**
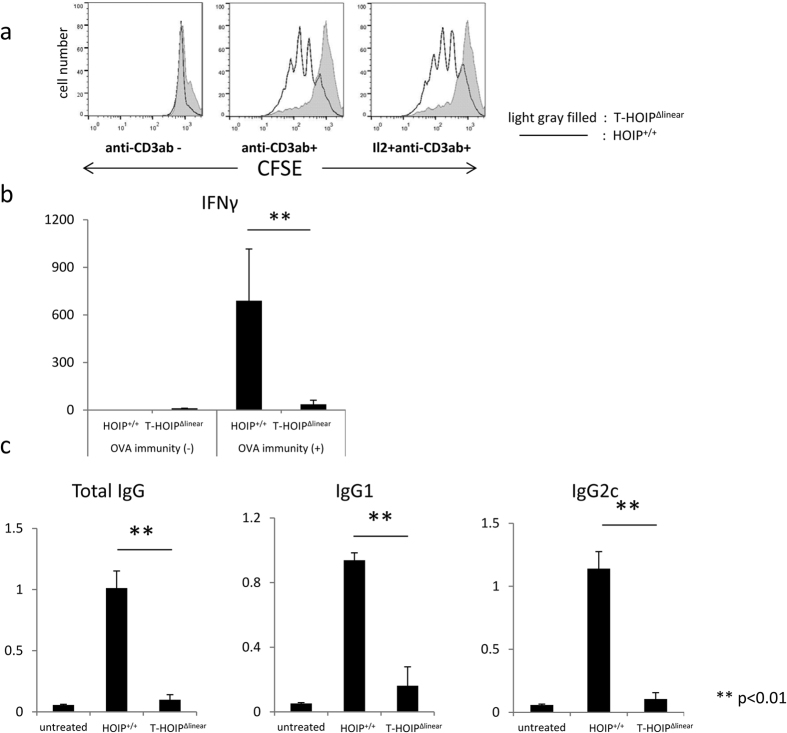
Impaired proliferation and cytokine secretion of CD4^+^ T cells in T-HOIP^Δlinear^ mice. (**a**) CFSE-labelled CD4^+^ T cells from spleens of control (line) or T-HOIP^Δlinear^ (filled gray) mice were stimulated for 3 days on plates coated with anti-CD3 mAb (1 μg/mL) in the absence or presence of recombinant IL-2 (10 U/mL). HOIP^+/+^ or T-HOIP^Δlinear^ mice at the age of 8 weeks were immunized by OVA protein (10 μg/mL) emulsified in CFA. (**b**) Serum IFN-γ was evaluated by ELISA ten days after immunization. Data show means ± SEM. **p < 0.01. (**c**) Serum anti-OVA IgG, IgG1 or IgG2c levels were evaluated by ELISA ten days after immunization. Data show means ± SEM. **p < 0.01. The data in these figures are representative of four independent experiments.

We evaluated the *in vitro* proliferative ability of CFSE-labeled CD4^+^ T cells from T-HOIP^Δlinear^ mice when stimulated by anti-CD3 mAb exposure. Those CD4^+^ T cells showed less CFSE dilution than did control cells, indicating relatively slower proliferative activity (Fig. 3a). The poorer proliferative activity of CD4^+^ T cells from T-HOIP^Δlinear^ mice was not rescued by the addition of IL-2 to the culture medium (Fig. 3a). To assess the role of HOIP in the functional differentiation of CD4^+^ T cells, we immunized T-HOIP^Δlinear^ mice and HOIP^+/+^ mice with OVA protein and evaluated anti-OVA specific immunoglobulin production and levels of serum cytokine 14 days after immunization. The CD4^+^ T cells from OVA-immunized T-HOIP^Δlinear^ mice produced lower amounts of IFN-γ than those from HOIP^+/+^ mice (Fig. 3b) and failed to produce anti-OVA-specific IgG, IgG1 and IgG2c (Fig. 3c).

### CD4^+^ T cells in T-HOIP^Δlinear^ mice failed to phosphorylate IκBα

**Figure 4:**
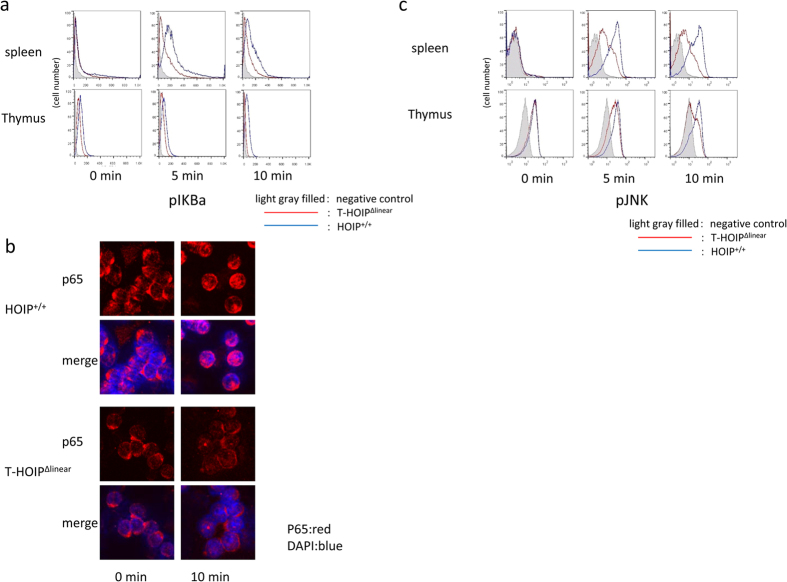
Impaired NF-κB and JNK activation in T-HOIP^Δlinear^ T cells. (**a**) Isolated CD4^+^ T cells from HOIP^+/+^ (blue) or T-HOIP^Δlinear^ (red) mice were stimulated with anti-CD3 mAb followed by anti-hamster IgG for the indicated time. The expression of phospho-IκBα was evaluated by flow cytometry. As the negative control, staining with isotype control IgG was used (filled gray). (**b**) Isolated CD4^+^ T cells from HOIP^+/+^ or T-HOIP^Δlinear^ mice were stimulated with anti-CD3 mAb followed by anti-hamster IgG for the indicated time. The expression of p65 (red) in CD4^+^ T cells ten min after stimulation were evaluated by confocal microscopy. The nucleus was stained with DAPI (blue). (**c**) Isolated CD4^+^ T cells from HOIP^+/+^ (blue) or T-HOIP^Δlinear^ (red) mice were stimulated with anti-CD3 mAb followed by anti-hamster IgG for the indicated time. The expression levels of phospho-JNK were evaluated by flow cytometry. As the negative control, staining with isotype control IgG was used (filled gray). The data in these figures are representative of four independent experiments.

We assessed the role of HOIP ligase in the activation of NF-κB in mature T cells. Thus, CD4^+^ T cells from T-HOIP^Δlinear^ mice and HOIP^+/+^ mice were stimulated by anti-CD3 mAb and phosphorylation of IκBα was evaluated. Anti-CD3 mAb treatment of CD4^+^ T cells from T-HOIP^Δlinear^ mice induced less phosphorylation of IκBα than observed in cells from control mice (Fig. 4a). We then analyzed nuclear translocation of NF-κB (p65) after anti-CD3 mAb-stimulation of CD4^+^ T cells from T-HOIP^Δlinear^ mice or HOIP^+/+^ mice. Little nuclear translocation of p65 was found in CD4^+^ T cells from T-HOIP^Δlinear^ mice compared with efficient translocation of p65 into the nucleus in control T cells (Fig. 4b).

The deficiency of HOIP ligase activity in B cells disturbs CD40 but not B cell receptor-mediated JNK activation^24^. Thus, we analyzed TCR-mediated JNK phosphorylation in T-HOIP^Δlinear^ mice T cells. Stimulation with anti-CD3 mAb induced less phosphorylation of JNK in HOIP ligase-deficient T cells compared with control cells (Fig. 4c). Those data demonstrated that the deficiency of HOIP ligase activity disturbed activation of not only the canonical NF-κB pathway but also the JNK pathway.

### T cells from HOIP-deficient mice lost viability

**Figure 5:**
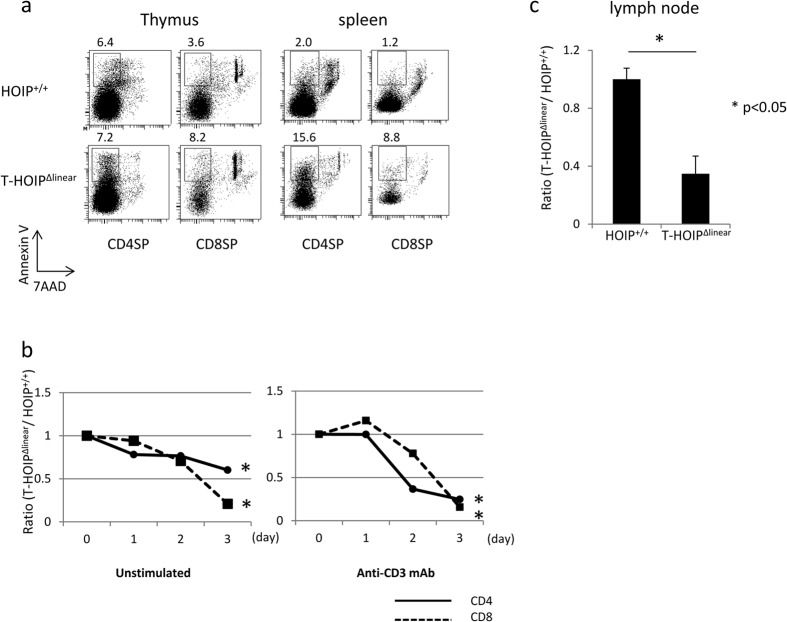
Impaired survival of T cells in T-HOIP^Δlinear^ mice. (**a**) Thymocytes and spleen cells were stained with anti-CD4 and anti-CD8 antibodies together with Annexin V and 7AAD. CD4^+^ or CD8^+^ T cells with an Annexin V^+^7AAD^−^ phenotype were evaluated. The number indicates the percentage of each population. (**b**) Isolated CD4^+^ or CD8^+^ T cells from HOIP^+/+^ or T-HOIP^Δlinear^ mice were cultured in the absence or presence of plate-coated anti-CD3 mAb. The cell number after the indicated number of days was counted. The value is calculated from the number of T-HOIP^Δlinear^/number of HOIP^+/+^ cells. *p < 0.05. (**c**) Isolated CD4^+^ T cells from HOIP^+/+^ (CD45.2) or T-HOIP^Δlinear^ mice (CD45.2) were transferred into nonirradiated B6 mice (CD45.1). The number of donor cells 7 days after transfer was counted. Data are shown as means ± SEM. *p < 0.05. The data in these figures are representative of five independent experiments.

We sought to assess whether the loss of mature T cells in T-HOIP^Δlinear^ mice was attributable to increased cell death. Thus, mature CD4^+^ or CD8^+^ T cells from the thymus and spleen were stained with Annexin V and 7AAD. Larger percentages of CD8^+^ T cells in the thymus and CD4^+^ or CD8^+^ T cells in the spleen of T-HOIP^Δlinear^ mice were positive for Annexin V than in control cells (Fig. 5a). Those data suggested that the deficiency of HOIP ligase activity increased the frequency of cell death in mature T cells, especially in early developmental stages of single positive cells in the thymus.

To confirm that CD4^+^ or CD8^+^ T cells from T-HOIP^Δlinear^ mice did not retain viability, we compared cell survival of CD4^+^ and CD8^+^ T cells from T-HOIP^Δlinear^ mice and HOIP^+/+^ mice. When CD4^+^ T cells from T-HOIP^Δlinear^ mice (CD45.2) and control (CD45.1) mice were cultured *in vitro* without any stimulation or after stimulation with anti-CD3 mAb, T cells from T-HOIP^Δlinear^ mice died more rapidly than those from HOIP^+/+^ mice (Fig. 5b). To determine if the impaired T cell survival also occurred *in vivo*, CD4^+^ T cells (CD45.2) from T-HOIP^Δlinear^ mice or HOIP^+/+^ mice were transferred into recipient C57BL/6 (CD45.1) mice. The number of CD4^+^ cells from T-HOIP^Δlinear^ mice was much lower than control cells 3 days after transfer into inguinal lymph nodes, (Fig. 5c). Those results suggested that CD4^+^ T cells from HOIP^−/−^ mice were prone to die compared with control CD4^+^ T cells.

### CD127 expression was lower in T cells from T-HOIP^Δlinear^ mice

**Figure 6:**
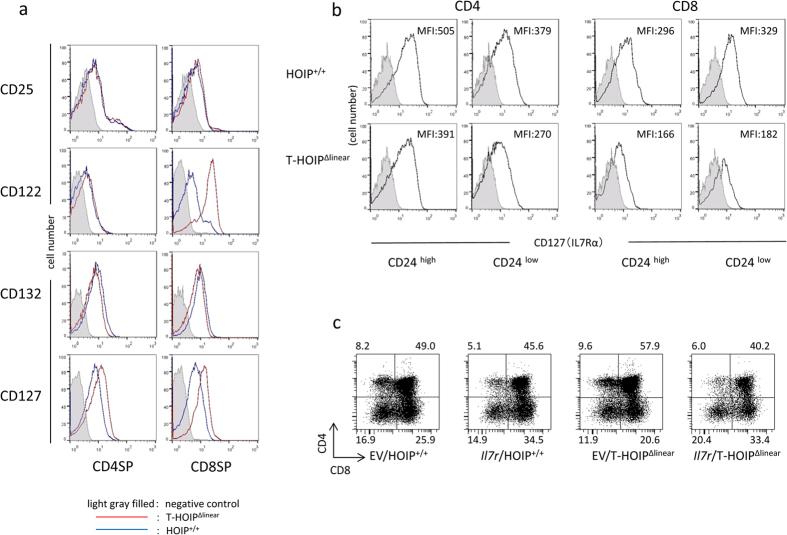
Defective IL-7Rα in thymocytes of T-HOIP^Δlinear^ mice. (**a**) Spleen cells from T-HOIP^Δlinear^ (red) or HOIP^+/+^ (black) mice were stained with anti-CD4, anti-CD8α, anti-CD25, anti-CD122, anti-CD127 and anti-CD132 antibodies. The expression of CD25, CD122, CD127 and CD132 in CD4^+^CD8^−^ (CD4SP) or CD4^−^CD8^+^ (CD8SP) was evaluated by flow cytometry. The negative control cells were stained with isotype controls (filled gray). (**b**) Thymocytes from T-HOIP^Δlinear^ or HOIP^+/+^ mice were stained with anti-CD4, anti-CD8α, anti-CD24 and anti-CD127 antibodies. The expression of CD127 by CD4^+^CD8^−^CD24^hi^ or CD4^+^CD8^−^CD24^low^, CD4^−^CD8^+^CD24^low^ or CD4^−^CD8^+^CD24^hi^ cells was evaluated by flow cytometry. As the negative control, cells were stained with isotype controls (filled gray). The number indicates the mean fluorescence intensity (MFI) of each population in the viable population. (**c**) Fetal thymocytes (day 15 fetal age) were infected with control retrovirus (EV) or retrovirus containing the CD127 gene (*Il7r*) and cultured in dGu-treated fetal thymus for 7 days. Thymocytes were stained with anti-CD4 and anti-CD8α antibodies and the expression gated on GFP^+^ cells was evaluated by flow cytometry. The number indicates the percentage of each population in the viable population. The data in these figures are representative of four independent experiments.

In order to determine the molecular mechanisms for impaired development of T cells from T-HOIP^Δlinear^ mice, we tested the expression of cytokine receptors on T cells. The expression levels of common γ-chain (CD132) were comparable between CD4^+^ and CD8^+^ T cells in T-HOIP^Δlinear^ mice and HOIP^+/+^ mice (Fig. 6a). However, the expression levels of IL-2Rα (CD25) and IL-2Rβ (CD122) were higher in splenic CD8^+^ T cells from T-HOIP^Δlinear^ mice. Moreover, the expression levels of IL-7Rα (CD127) were relatively high in CD4^+^ and CD8^+^ splenic T cells from T-HOIP^Δlinear^ mice (Fig. 6a). In contrast, the expression of CD127 was lower in thymic CD4^+^ and CD8^+^ T cells from T-HOIP^Δlinear^ mice (Fig. 6b).

*Il7r* (CD127) was reported to be a target gene for NF-κB signaling^25^. As IL-7 is required for CD8^+^ T cell survival, we examined if impaired development of T cells from T-HOIP^Δlinear^ mice was attributable to low CD127 expression. CD127-encoding retrovirus was infected in fetal thymocytes from T-HOIP^Δlinear^ mice. The GFP-expressing thymocytes were cultured in fetal thymus for 7 days and the development of mature T cells was examined. The overexpression of CD127 increased the frequency of mature CD8^+^TCRβ^+^ but not CD4^+^TCRβ^+^ T cells (Fig. 6c). Those data indicated that impaired CD8^+^ T cell survival in T-HOIP^Δlinear^ mice is, at least partly, attributable to low CD127 expression.

## Discussion

LUBAC-mediated poly-linear ubiquitination is a crucial event for activating the NF-κB pathway^21^,^20^. However, the roles of LUBAC-mediated NF-κB regulation in T cell activation or in development have been unresolved. In this paper, we show that among the LUBAC components, HOIP ligase activity is required for the development of mature T cells and *is crucial for CD4*^*+*^ *T cell proliferation.* T-HOIP^Δlinear^ mice T cells failed to upregulate CD127, which was attributable to the impaired survival of thymic CD8^+^ T cells but not CD4^+^ T cells in T-HOIP^Δlinear^ mice. These findings demonstrate the crucial contribution of HOIP-mediated linear ubiquitination of NEMO to T cell development. They support a model in which CD4^+^ and CD8^+^ T cells have distinct molecular requirements for NF-κB-mediated molecules downstream.

T cell development in the thymus is controlled by a multistep process utilizing the TCR, costimulatory molecules and cytokine signals, each of which is required during specific stages of development. Given that the TCR and cytokines signaling are crucial for T cell development, with NF-κB downstream for various receptors in conventional T cells, HOIP could control thymic T cell differentiation at multiple points. Our data demonstrated that mature CD4^+^ or CD8^+^ T cells were markedly diminished with reduced expression of CD127 in T-HOIP^Δlinear^ mice, a deficit that was rescued by overexpressing CD127 on CD8^+^ T cells, at least in an *in vitro* culture system. IL-7 functions in the survival and development of conventional CD4^+^ and CD8^+^ T cells, as evidenced by a markedly reduced number of mature CD4^+^ and CD8^+^ T cells in CD127-deficient mice^15^. Therefore, the impaired survival of CD8^+^ T cells in T-HOIP^Δlinear^ mice could be, at least partially, attributable to the reduced expression of CD127. In contrast, the development of CD4^+^ T cells could not be rescued by overexpressing CD127, suggesting that the dysregulation of other target molecules downstream from HOIP is responsible for the impaired survival of CD4^+^ T cells. Those data suggest a model in which CD4 and CD8 T cells require distinct regulation of target molecules downstream of HOIP for their survival in the thymus.

HOIP complexes with HOIL-1L and SHARPIN^26^,^22^. Mutations in the murine *Sharpin* gene cause spontaneous *chronic proliferative dermatitis (cpdm*) that develops into psoriasis-like proliferative skin lesions, splenomegaly, absence of Peyer’s patches and low levels of serum immunoglobulin^27^. A recent study reported that patients with a loss-of-function mutation in HOIL-1L suffered from chronic autoinflammation, invasive bacterial infections and muscular amylopectinosis^28^. Furthermore, an inherited mutation in HOIP causes multi-organ autoinflammation, combined immunodeficiency, subclinical amylopectinosis, and systemic lymphangiectasia^23^. These findings suggest a distinct requirement for each LUBAC subunit to control downstream pathways. In contrast to the autoinflammatory phenotypes associated with HOIP- or HOIL-1L-deficiency in humans, the present study revealed that a deficiency in HOIP ligase activity impaired NF-κB activation leading to the impairment of both CD4^+^ and CD8^+^ T cell development without any inflammatory responses. As LUBAC ligase activity was deleted only in T cells in our mouse study, the loss of function of non-T cells might be involved in the development of inflammatory responses.

The CBM (CARMA1–Bcl10–Malt1) TCR adaptor complex regulates TCR-dependent NF-κB activation^29^,^30^,^31^. Despite the important roles of CARMA1 in NEMO activation, CARMA1-deficient mice have normal T-cell development and normal peripheral T-cell numbers and ratios^32^,^33^. However, they do have a defect in the development of intrathymic CD4^+^CD25^+^ regulatory T cells^34^. In contrast, T cell-specific, NEMO-deficient mice are devoid of mature CD4^+^ and CD8^+^ T cells in the thymus^18^, a finding that is similar to T cell-specific, HOIP ligase activity deficient mice. Furthermore, HOI -ligase activity deficient CD4^+^ T cells have a defect in TCR-mediated proliferation and NF-κB activation. Those results suggest that the engagement of TCR activates NEMO by utilizing CARMA1-dependent or -independent pathways, and that LUBAC-mediated linear ubiquitination of NEMO through engagement of TCR is essential for the survival of mature T cells. In addition, a recent paper revealed that LUBAC integrates the CBM complex and that NF-κB reporter activity is stimulated following antigen receptor ligation independent of its catalytic activity^35^. However as this study was performed by evaluating NF-κB reporter activity in Jurkat cells that had been transfected with siRNA against HOIP and siRNA resistant ligase activity-inactive HOIP, the effect of the residual activity of endogenous HOIP might not be negligible. In future studies, it will be necessary to evaluate which domains of HOIP are crucial for binding with the CBM complex.

In this report, we found that canonical NF-κB signaling through linear ubiquitination by LUBAC was an essential molecular pathway that regulated CD4^+^ and CD8^+^ T cell development. Our data highlight a previously unknown molecular link between LUBAC and mature CD4^+^ and CD8^+^ T cell survival. Those data also suggest new approaches for inhibiting HOIP ligase activity and thereby suppressing T-cell-mediated immune responses.

## Methods

### Mice

Six- to 8-week-old C57BL/6 mice were purchased from Japan SLC (Hamamatsu, Japan). *Rnf31*^Δlinear/Δlinear^ mice were previously described^24^. C57BL/6 mice (CD45.1) and CD4-*Cre* transgenic mice were purchased from Jackson Laboratory (MA, USA). All animal experiments were approved by an animal research ethical committee of Tokushima University and were performed according to its guidelines.

### Flow cytometric analysis

The livers were homogenized and resuspended in gradient buffer that contained 2.5% FBS plus 10 mL Percoll (GE Healthcare) and 2 mL Alsever’s solution (Sigma-Aldrich). After centrifuging the solution for 20 min at 2000 rpm at room temperature, the upper layer was discarded. To isolate lymphocytes, the lower layer that contained lymphocytes and RBC was hemolyzed with NH_4_Cl and washed with PBS. Thymocytes, splenocytes or lymph nodes cells were filtered through a 100 μm mesh. Fluorochrome-conjugated monoclonal antibodies specific for mouse CD8α (53–6.7), CD44 (IM7) and Foxp3 (3G3) were purchased from Tonbo Biosciences (San Diego, CA, USA). Antibodies specific for CD4 (GK1.5), CD122 (TMβ1), B220 (RA3-6B2) and TCRγ (GL3) were obtained from BD Biosciences (*Franklin Lakes*, NJ, USA). Antibodies specific for CD24 (M1/69), CD25 (3C7), CD45.1 (A20), CD45.2 (104), CD62L (MEL-14), CD69 (H1.2F3), CD127 (A7R34), CD132 (TUGm2), NK1.1 (PK136), and TCRβ (H57-597) were bought from BioLegend (San Diego, CA, USA). The CD1d tetramer was provided from the NIH tetramer facility. Antibodies specific for phospho-IκBα (39A1431) was purchased from Abcam (Cambridge, UK). Antibodies specific for phospho-JNK (G9) were purchased from Cell Signaling Technology (Danvers, MA, USA). All samples were resuspended in PBS staining buffer containing 2% FBS and 0.01% NaN_3_, and pre-incubated for 15 min at 4 °C with 2.4G2 supernatant to block Fc receptor, then washed and stained with specific mAbs for 20 min at 4 °C. For intracellular staining, cells were fixed with 4% paraformaldehyde and permeabilized with 0.01% saponin-containing buffer. Data were collected on a FACSCanto II (BD Biosciences) and analyzed using FACS Diva (BD Biosciences) or FlowJo (Tree Star, OR, USA) software.

### T cell proliferation analysis

CD4^+^ T cells were isolated with anti-CD4 microbeads (Miltenyi Biotech, Germany) and labeled with CFSE (10 mg/mL). CD4^+^ T cells (3 × 10^6^/well; 24-well plates) were stimulated with plate-coated anti-CD3 mAb (145-2C11) (1 μg/mL) (Tonbo Biosciences) in the absence or presence of mouse recombinant IL-2 (10 U/mL) (Miltenyi Biotec) for 3 days.

### OVA immunization

Mice were immunized with OVA protein (50 μg) emulsified in CFA (Sigma, Saint Louis, MO, USA) and the titers of OVA-specific antibodies (IgG, IgG1 and IgG2c) were measured by ELISA using HRP-conjugated anti-mouse IgG, IgG1 or IgG2c (Southern Biotech, Alabama, USA) as the secondary antibodies.

### T cell survival

Purified CD4^+^ T cells (4 × 10^6^) from C57BL/6 (CD45.1) and HOIP^−/−^ (CD45.2) mice were cultured without any stimulation or stimulated with plate-coated anti-CD3 mAb (1 μg/mL). After culturing cells, cell number was counted, cells were stained with anti-CD4, CD45.1 and CD45.2 antibodies and the number of cells in each population was calculated.

### ELISA

ELISA for IFN-γ was performed using an ELISA kit from eBioscience.

### Confocal laser-scanning microscopy analyses

T cells were isolated with a pan-T cell isolation kit (Miltenyi Biotec) and stimulated for 10 min at 37 °C with anti-CD3 mAb followed by anti-hamster IgG. Cells were then seeded on poly-l-lysine hydrobromide-coated cover glass, fixed with 4% paraformaldehyde and permeabilized with acetone. Staining with anti-p65 mAb (1 μg/mL) (Santa Cruz Biotech) was followed by Alexa Fluor 546-conjugated goat anti-rabbit IgG (Invitrogen). The nucleus was stained with DAPI. The observations were performed using an FV10i confocal microscope (OLYMPUS, Japan). Several cells were analyzed for each labeling condition, and representative results are presented.

### Fetal thymic organ culture

Fetal thymus (fetal age, day 15) from C57BL/6 mice was cultured in the presence of deoxyguanosine (1.35 mM) (Sigma) on Transwell plates for 7 days. Fetal thymocytes (fetal age, day 15) from HOIP^+/+^ or HOIP^−/−^ mice were isolated and infected with control retrovirus or *Il7r*-encoding-retrovirus as previously reported^36^. The pKE004 retrovirus vector^37^ that encodes IRES-GFP and *Il7r* was transfected into Plat-E cells^38^ to generate retrovirus. The infected thymocytes were cultured for one day in the presence of IL-7 (5 ng/mL) (eBioscience). Thymocytes were hanging-drop cultured with deoxyguanosine-treated thymus using a Terasaki plate for one day. Then the thymus was cultured on a Transwell plate for 7 days.

### Statistical analysis

For all experiments, the significance of differences between groups was calculated using the Mann-Whitney U test for unpaired data. Differences were considered significant when *p* < 0.05.

## Additional Information

**How to cite this article**: Okamura, K. *et al.* Survival of mature T cells depends on signaling through HOIP. *Sci. Rep.* **6**, 36135; doi: 10.1038/srep36135 (2016).

**Publisher’s note:** Springer Nature remains neutral with regard to jurisdictional claims in published maps and institutional affiliations.
